# Embryonic cerebrospinal fluid in brain development: neural progenitor control

**DOI:** 10.3325/cmj.2014.55.299

**Published:** 2014-08

**Authors:** Angel Gato, M. Isabel Alonso, Cristina Martín, Estela Carnicero, José Antonio Moro, Aníbal De la Mano, José M. F. Fernández, Francisco Lamus, Mary E. Desmond

**Affiliations:** 1 Departament of Anatomy and Radiology, Faculty of Medicine, Valladolid University, Valladolid, Spain; 2 Laboratory of Nervous System Development and Teratology, Institute of Neurosciences of Castilla y León (INCYL), Valladolid University, Valladolid, Spain; 3 Departament of Cellular Biology, Histology and Pharmacology, Faculty of Medicine, Valladolid University, Valladolid, Spain; 4 Department of Biology, Villanova University, Villanova, PA, USA

## Abstract

Due to the effort of several research teams across the world, today we have a solid base of knowledge on the liquid contained in the brain cavities, its composition, and biological roles. Although the cerebrospinal fluid (CSF) is among the most relevant parts of the central nervous system from the physiological point of view, it seems that it is not a permanent and stable entity because its composition and biological properties evolve across life. So, we can talk about different CSFs during the vertebrate life span. In this review, we focus on the CSF in an interesting period, early in vertebrate development before the formation of the choroid plexus. This specific entity is called “embryonic CSF.” Based on the structure of the compartment, CSF composition, origin and circulation, and its interaction with neuroepithelial precursor cells (the target cells) we can conclude that embryonic CSF is different from the CSF in later developmental stages and from the adult CSF. This article presents arguments that support the singularity of the embryonic CSF, mainly focusing on its influence on neural precursor behavior during development and in adult life.

## Singular characteristics of embryonic CSF

**Figure 1:**
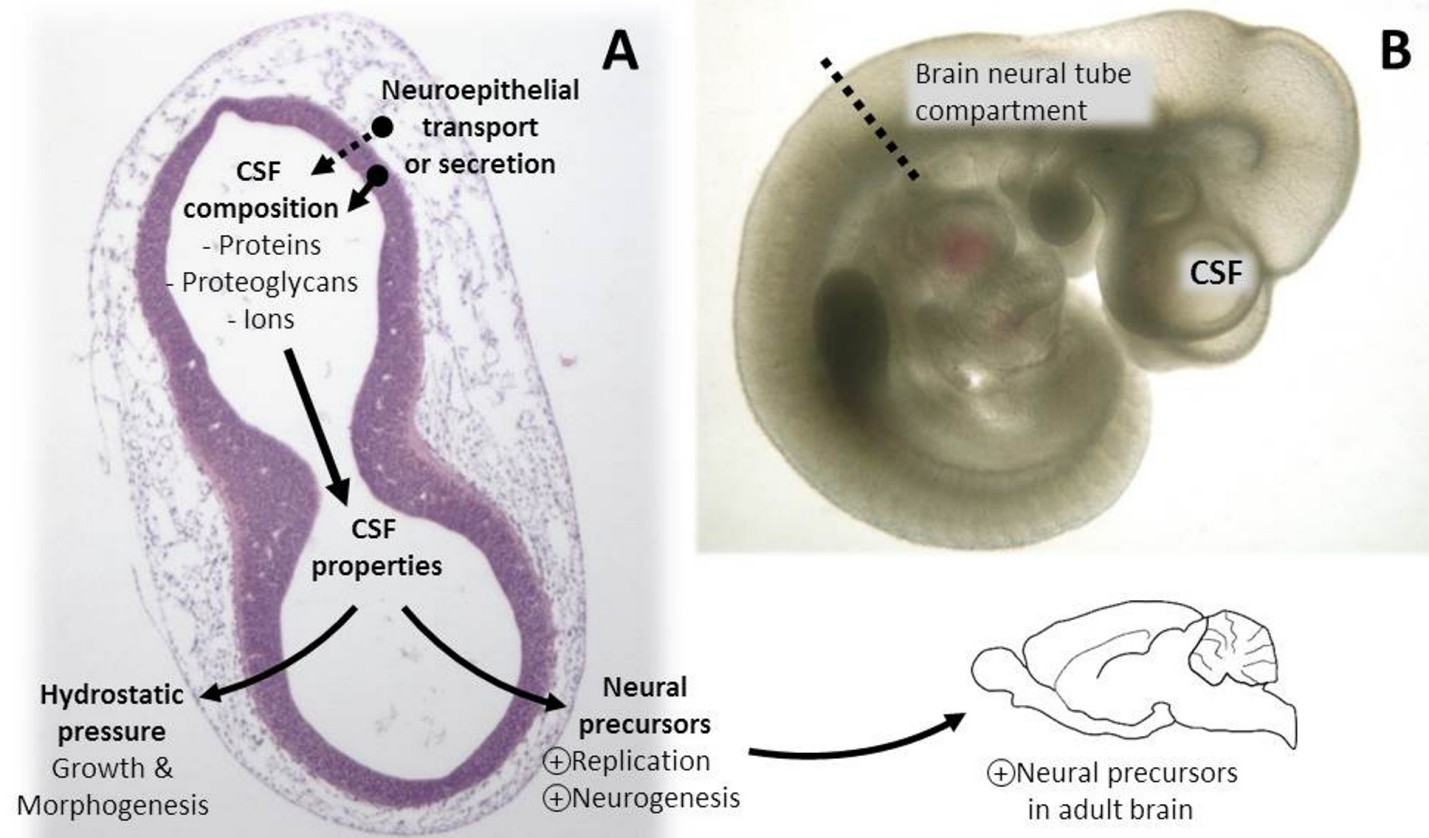
Development of the mouse embryo after 10.5 days. (**A**) Transversal histological hematoxilin-eosin stained section. (**B**) Macroscopic view showing the neural tube by transilumination.

We will first discuss what makes embryonic CSF different from fetal and adult CSF. According to the classic concept, which applies to the fetal and adult periods, CSF fills the cavities of the ventricular system and subarachnoid space. It is in direct contact with different cellular types such as the ventricular layer in the ventricular system and the pial layer in the subarachnoid space, as well as with specific cellular populations such as the choroid plexus cells and the subventricular organs. In this period, CSF is enclosed in a non-distensible cavity system with permanent production, circulation, and reabsorption (Figure 1).

At the earliest stages of brain development, embryonic CSF fills a brain restricted cavity, which is closed and undergoes a quick change in volume and morphology. Embryonic CSF is located in a cavity in the anterior part of the neural tube, the brain anlagen where we can clearly differentiate three major parts of the brain (anterior brain, midbrain, and hindbrain), which evolve quickly (1). This cavity is surrounded by a unique and specific type of cells called neuroepithelial precursors. There are no specific cellular populations involved in the secretion and reabsorption of the fluid and it does not appear to circulate. This period begins with the formation of the neural tube as a result of a morphologically complex process known as neurulation (2,3). The critical point, which marks the beginning of this process, is the closure of the anterior neuropore and the subsequent medullar collapse of the cervical part of the neural tube. Together they make a closed cavity system inside the brain anlagen (4,5) in which CSF and neuroepithelium function interdependently. The end of the embryonic CSF period is marked by two processes: the appearance of the choroid plexus anlagen (6,7), which is a new CSF production center, and the opening of the rombencephalic roof, an area involved in communication with the mesenchyme, where the subarachnoid space will be developed. This transitory, specific situation that lasts for a short period of time is a relevant period in brain development because it includes a very intense replication process leading to the neural precursor population expansion, as well as the beginning of neuronal precursor differentiation named “neurogenesis,” a process that suddenly becomes very intense (1).

Regarding embryonic CSF composition, many studies in different species found proteins to be the most important components of CSF during embryonic and fetal development (Figure 1). For example, CSF in chicken embryos has a thirty times higher protein concentration than in adult chickens (8,9). Another interesting point is that chicken and sheep embryonic and fetal CSF protein concentration increases progressively until the end of the fetal stage (10-12), while in rats it remains elevated until after birth (13). In all cases, the protein concentration after birth falls dramatically until it reaches the adult values. High CSF concentration of proteins such as albumin, fetuin, alpha-fetoprotein, transferrin, and lipoproteins has been demonstrated during the early fetal stage in sheep (9,10) and rats (13). In rats, alpha-fetoprotein and albumin account for more than 50% of the total protein content. Gato et al (12) used SDS-PAGE electrophoresis to analyze the entire CSF protein composition at the earliest stages of development in chicken and rat embryos showing 21 different protein fractions. In the last decade, proteomic analysis of embryonic, fetal, and adult CSF in different species resulted in identification of many proteins, including extracellular matrix, enzymes, proteoglycans, apolipoproteins, and growth factors and cytokines, showing a complex composition pattern, which confirms the relevant biological role of this fluid (14-16). Taken together, these studies suggest a common protein pattern in the CSF but with striking differences during the ontogeny, which can explain the different roles of CSF during the life span.

Increase in the accuracy and sensitivity of the proteomic techniques allowed the analysis of the complete molecular composition of the CSF in different species and at different stages of development. Such studies are necessary to assess the CSF usefulness in therapeutic strategies.

Another specificity of the embryonic CSF is that it is confined in a restricted space. In fetal and adult stages, the CSF is located in the brain cavities or ventricles, medullar cavity, and subarachnoid space. However, at the earliest developmental stages, there is no subarachnoid space in the mesenchyme and no functional communication between the brain cavity and the space outside. Consequently, embryonic CSF is restricted to the cavity of the brain vesicles. A specificity of the neural tube in these stages is that the cavity that encloses the CSF is completely surrounded and sealed by the apical end of the homogeneous population of neuroepithelial precursors, leading us to two conclusions: 1) the origin of the CSF must necessarily be mediated by neuroepithelial precursors and 2) neuroepithelial precursors must be the target cells.

Regarding the first point, the content of the sealed cavity must come from the cavity wall, which consists of the brain neuroepithelium. Two different mechanisms of the CSF origin have been proposed (17): the first is apical secretion of specific macromolecules by neuroepithelial cells into the cavity. This mechanism is mainly supported by the studies of Gato et al (18-20), which show an active apical secretion of chondroitin sulfate proteoglycan. The second is an active intercellular or intracellular transport of molecules, such as FGF2 (21) and other types of proteins (17,22) or ions (23) across the neuroepithelium from the basal to apical side. Some studies suggest that there are specific areas where brain neuroepithelial transport takes place, which is similar to a primitive choroid plexus (17).

On the basis of these data, we can conclude that embryonic CSF can be a means of inner communication between neuroepithelial precursor cells, in which some populations are involved in the creation of a complex signaling fluid, while other answer these signals.

## Specific roles of embryonic CSF: the interaction with the neural precursors during development and in the adult brain

Here we will try to review the specific roles attributed to the embryonic CSF during early brain development. As we have stated before, one of the main characteristics of embryonic CSF is the permanent interaction with the apical end of the neuroepithelial precursors, which can be considered brain stem cells during development (Figure 1). Today we know that the cellular behavior of these precursors at the earliest stages of development highly depends on the physicochemical properties of CSF, supporting the hypothesis that the neuroepithelium and CSF are interdependent in the developing brain. In this respect, embryonic CSF has two roles:

The first is that embryonic CSF creates an expansive force inside the brain cavity, involved in the generation and regulation of the brain anlagen growth and morphogenesis. As we have already said, in the earliest stages of development, the brain cavity becomes a sealed system filled by a fluid that exerts positive pressure against the neuroepithelial wall. This positive pressure is necessary for the brain’s expansive growth both in the cavity and neuroepithelial volume and also for the normal morphogenesis (24,25). This mechanism is also necessary for the establishment of primary vesicular pattern in the brain, mainly based on the existence of the regional growth differences in the cephalic neural tube (1).

Regulation of brain growth and morphogenesis by embryonic CSF has been partially explained in the sense that it is a physical mechanism that creates a positive hydrostatic pressure inside the sealed cavity against the neuroepithelial wall, which has local growth differences. This mechanism is created and developmentally regulated by neuroepithelial precursor cells synthesizing and apically secreting into the brain cavity osmotically active molecules such as proteoglycans, together with a simultaneous and specific ionic transport across the neuroepithelium. Proteoglycans are responsible for trapping of water inside the cavity, which generates the hydrostatic pressure (19,20,23,26). The expansive force inside the cavity must be coordinated with the growth of the neuroepithelial wall to generate not only volumetric growth but also differential growth, which drives morphogenesis and brain regionalization. Further research should investigate how this osmotic mechanism is regulated during development and how it is coordinated with neuroepithelial growth in order to contribute to normal brain development. A recent study (27) made a step in this direction by proposing focal adhesion kinases (FAKs) as a link between CSF pressure and neuroepithelial precursors replication.

The second mechanism is the regulation of basic cellular behavior of brain neuroepithelial precursors by embryonic CSF. When Desmond and Jacobson (24) showed a decrease in the tissue volume of the brain neuroepithelium after an experimentally induced loss of embryonic CSF, our group started to investigate if there was a direct relation between CSF and the basic behavior of neuroepithelial cell precursors (28). We demonstrated that besides the previously described physical influence on brain development, CSF exerted a “biological” influence, which is essential to regulate the key functions of brain neuroepithelial precursors such as cell survival, replication, and neuronal differentiation. We showed this influence by developing a neuroepithelial culture technique that allowed the exposure or deprivation of neuroepithelial tissue (28,29). The main conclusion of these studies is that neuroepithelial precursors are not self-sufficient and need the influence of embryonic CSF to develop a normal behavior pattern.

After these studies, we focused on the identification of the CSF molecules responsible for these trophic properties. To date, several individual factors have been found to be involved in each mechanism, ie, survival, replication, and differentiation. Many studies have focused on the identification of mitogenic factors in CSF, such as FGF2, IGF1, NGF, and EGF (29-31), which regulate the mitogenic activity of neuroepithelial precursors at different stages of development. However, other components of embryonic CSF, such as retinol and retinol binding protein, involved in the regulation of the synthesis of retinoic acid by some specific cell population in the neuroepithelium, have been shown to be key factors in neuronal differentiation of neuroepithelial precursors (32-34). However, more CSF components with specific roles in brain development probably still need to be discovered.

On the other hand, it was overlooked that embryonic CSF had a role as an activator of neurogenesis in the adult brain. Surprisingly, neural precursors have been shown to have “astrocytic” nature in the subventricular zone (SVZ) and in the dentate gyrus (DG) of the hippocampus in adult mammals (35). Despite the same cellular lineage, across the life span they show different status (36,37) – those from the embryonic stages persist into the fetal stages as radial glial cells, and in some places such as the SVZ and DG, radial glia persist as a particular type of astrocytes that preserve the precursor characteristics, self renewing to expand the population and pluripotentiality to differentiate into glia and neurons (Figure 1).

These data lead us to the key question: Why does the same cellular lineage result in a quite different behavior at different life stages? In fact, the main difference between the embryonic and the adult stages is the intensity of activity rate, which is at its maximum in the embryonic stages and shows a permanent decrease later on, leading to a restricted regenerative ability.

In the last decades, many research groups have reported that at any life stage the degree of neural precursor activity is determined by the “cellular niche,” which is defined as the surroundings of the neural precursor cells and includes the mature cells (neurons and glia), immature cells generated by the precursors, extracellular matrix and all types of intercellular signals, including those from the microvessels, and the fluid content in the ventricular system (38-40). The intercellular signals involved in the niche concept have been the subject of many studies focusing on individual growth factors, transcription factors, cytokines, and morphogens. They all suggest a complex situation in which many different factors play similar roles (mainly mitogenic and/or neurogenic) at the same time, overlapped or at different stages. There is a group of niche-specific signals that remain stable across the life span, but other signal components seem to be specific for each stage – embryonic, fetal, and adult (40), supporting the idea that the specific neural precursor activity rate at each ontogenic stage could be a result of a specific composition of the niche signals. A more comprehensive explanation is given in specific reviews published in the last years (38).

Finally, literature data support the fact that the CSF is a part of the niche. In this way, neural precursors are in permanent and direct contact with the CSF content in the brain cavities, which directly influences the precursor behavior and is consequently considered a source of instructive signals that play a key role in the niche activity (31,41,42). In this context, it has been stated that CSF composition shows many ontogenic and phylogenic differences in different developmental stages (43,44) and that it could be different in a particular stage in each brain ventricle, conditioned by the specific secretion of each choroid plexus (45). These data support the idea that in its development from embryo to adult stage CSF becomes less and less neurogenic, confirming the apparently contradictory results (46,47) that described mitogenic but also gliogenic inductive effect of adult CSF on adult brain neural precursors, and our recent results (41) showing mitogenic but also neurogenic inductive effect of embryonic CSF on adult brain neural precursors.

In conclusion, embryonic CSF plays a key role in brain growth by creating a hydrostatic pressure inside the brain anlagen cavity. Furthermore, it is a powerful mitogenic and neurogenic inductor in neural progenitors at the earliest stages of development, when the activity of neural precursors is at its maximum. Neural progenitors in adult mammal brain remain responsive to the embryonic factors present in embryonic CSF, which increases the niche activity and enables the use of embryonic CSF-specific factors as a tool to induce brain neuroregeneration.
